# Inquiries into the Healthy and Diseased Appearances of the Mucous Membrane of the Stomach and Intestines

**Published:** 1828-04

**Authors:** W. E. Horner

**Affiliations:** adjunct Professor of Anatomy in the University of Pennsylvania.


### STOMACH AND INTESTINES.

*Inquiries into the Healthy and Diseased Appearances of the Mucous Membrane of the Stomach and Intestines.* By W. E. Horner, M.D. adjunct Professor of Anatomy in the University of Pennsylvania.

(Concluded from page 217.)

In speaking with Dr. Physick on the affections of the stomach, he told me that his experience led him to think that the highest grades of its irritation were attended neither by pain nor vomiting. The state of inflammation is so exalted, that its effects approximate those of the most deleterious poisons, which cause sudden death without local pain, fever, or any very sensible derangement of the functions, except mere weakness and a sense of illness. The following instance will illustrate this:

*Observation 6th.—Acute Gastritis.*—Henry Turner, a black man, aged fifty-five, assistant in the apothecary's shop at the Alms-house, after an apparently slight indisposition of a few days, which seemed to require rest rather than any thing else, while sitting up in his bed, suddenly expired almost without a struggle, May 4th, 1827. On dissection, twenty-four hours afterwards, the stomach was found of middle size, thick and dense, its mucous coat thrown into numerous, well-marked, elevated rugæ, and almost universally of a deep arterial red; the red globules of blood were extravasated in numerous spots and blotches in the thickness of the mucous coat and along the summits of the rugæ. This was a patient of Dr. Hodge, and I witnessed the dissection by chance. I declared unhesitatingly that the disease was an exasperated inflammation of the stomach, but, owing to the want of an assignable cause for it, as well as the want of appropriate symptoms during life, the opinion was not very readily acquiesced in. In a few days afterwards it was ascertained, through Mr. Marks, the apothecary at the Alms-house, that Turner had been taking private draughts from the bottle of Hoffman's Anodyne: at least, the quantity missing could be accounted for in no other way.

*Observation 7th.—Chronic Peritonitis and Gastro-enteritis.*—Alms-house, July 26th, 1827. Examination twelve hours after death.

John Neaman, aged seventy-seven, has been for two years a resident of this house. For the last nine weeks has complained of want of appetite, a sense of fulness over the abdomen, and pain in that region, but not so severe as to make him complain, or disquiet him much. Has had no diarrhœa, no vomiting, and no fever which called the attention of persons around. Eight days ago, the little food which he was in the habit of taking produced such a sense of fulness and distress that he determined to take no more, or at least the smallest possible quantity: since then he has taken a little bread, and now and then a spoonful of milk. His complaints being obscure, there has been no medical treatment within these eight days, and scarcely any before. At a distant time, which is not precisely remembered, some tincture of bark was administered by one of the young men of the house. He died seemingly from exhaustion.

Exterior habit: Skin pallid as usual after death; marasmus not exceeding that common at his time of life.

Abdomen: Peritoneum rough, with universal small miliary bodies, resembling tubercles, thickened somewhat by a coating of coagulating lymph, continuous with the tubercles. Peritoneum, on abdominal muscles and also on diaphragm, interspersed with patches of red blood; small coagula of blood adhering to its interior in many places, and abundant points of the same, as if from ruptured vessels; its cavity contained about three quarts of fluid, half serum and half blood blended, coagula of blood floating in this mixture. Mesentery studded at distant intervals with these small points of blood.

Stomach: Exterior or peritoneal aspect, pallid and healthy. Internal condition—contained no food, and a little mucus tinged with bile; cardiac termination of mucous coat of œsophagus of a deep red, from distinctly injected vessels; the whole mucous coat interspersed at somewhat distant intervals with small black spots of extravasated blood, from half a line to one line in diameter: for three inches around the pyloric orifice, these spots were more abundant and larger. Small intestine: mucous coat healthy, but the peritoneal coat of the colour of lampblack in many parts, and more or less of a sooty hue in its whole length. Large intestine: exterior or peritoneal face healthy, but dull from the disorganization beneath it. Mucous coat covered with black patches of blood extravasated in its thickness and beneath it: it resembled very much the spotted appearance of some of the sea-shells, and contained a small quantity of hardened fæces; small miliary bodies, like those on the peritoneum, abounding in it, but I could not distinguish whether they were mucous glands, or such tubercles as existed on the peritoneum. Liver, small and flabby. Spleen, natural.

The following are examples that redness is not found invariably in those exalted irritations of the stomach producing immediate death.

*Observation 8th.—Gastric Irritation.*—Jan. 28th, 1827, the daughter (aged eight months) of Mrs. A. D., without previous indisposition, and while playing in apparently excellent health on the lap of her aunt, was taken with a sudden spasm, and died instantly. This occurred after a hearty draught of cow's milk, on which the child was accustomed to feed.

The day following I made an examination, twenty-four hours after death, with Dr. James, the family physician, and the following appearances were presented:

Head: The flat bones much tinged with congested blood; the veins of the pia mater turgid. Serum under tunica arachnoidea; a drachm of serum in the right ventricle of the brain.

Thorax: Every thing natural.

Abdomen: About one gill of a cheese-like coagulum of milk in the stomach. The serous part had been continually running out of the mouth and nostrils since the death of the child, so as to require the constant use of cloths to soak it up; the quantity, therefore, could not be rigidly estimated, but probably did not fall short of two gills. The mucous coat of the stomach was not vascular, but of a pearl colour.

All the mesenteric glands were very much enlarged, but had nothing of a scrofulous consistence or texture.

The child, owing to the ill health of the mother, had been weaned at the age of one month, and brought up on cow's milk. That the coagulum formed from the latter was indigestible, perhaps from its quantity, is proved by the fact that pieces of it, tinged slightly with bile, were found in the lower part of the jejunum. It is pretty clear in this case that the undue quantity of coagulum in the stomach was the cause of death.

*Observation 9th*, March 29th, 1826.—*Gastric Irritation.*—Yesterday the infant daughter (aged ten months) of Mr. W. H. was seized, in a short time after eating half a table-spoonful of rice-pudding, with convulsions. The insensibility was complete, and the spasm universal; respiration was carried on with the greatest difficulty, irregularity, and interruption. This was at half-past four o'clock P.M. Happening to pass the house almost at that instant, my assistance was requested. In a few minutes, Drs. Hodge and Griffith arrived.

A warm bath was immediately resorted to. Three grains of tartar emetic were dissolved in a wine-glassful of water, and administered by the tea-spoonful every five or ten minutes. The attempt to raise a vein in the arm being ineffectual, I opened on the right side of the head three superficial veins, and on the left side two, along with a branch of the temporal artery. From these several openings about three and a half ounces of blood were drawn. About three-fourths of an hour were thus consumed, when the convulsions ceased. As the infant was teething, the gums were then cut with a lancet.

The child was an unusually fine one; had, to the moment of illness, enjoyed most excellent health, and had, indeed, been out on a visit to its friends that very morning. The indication seemed, therefore, sufficiently marked that the rice-pudding, along with a prune which had been eaten in the morning, was the cause of the convulsions, and that these matters, if possible, should be expelled from the stomach.

As, notwithstanding the cessation of the convulsions, the insensibility and difficult respiration still remained without much mitigation, about twenty-five grains of Pulv. Ipecac. in three tea-spoonsful of brandy, were administered in divided doses, at small intervals of time. This not producing the desired effect, a tea-spoonful of powdered mustard was given by portions, which also failing, twelve grains of Sulphate of Zinc were next given in divisions. This also failing, we next resorted to tickling the fauces with a finger, and to the introduction of a feather down the œsophagus, almost to the cardiac orifice of the stomach, some five or six times: still there was no vomiting induced. These several trials occupied until eight P.M. when the child revived very much, and was able to sit up.

It should also be mentioned that sinapisms had been, during our efforts, applied to the soles of the feet and to the epigastrium; and that two or three injections of spirits of turpentine had been administered, with the effect of purging as often.

About ten o'clock P.M. the infant seemed to recognise persons, smiled on being addressed in a soothing, playful manner, and afforded a prospect of recovery. At half-past twelve o'clock at night, after a slight convulsion, it died.

*Autopsy*, seventeen hours after death, the weather being moderate, and no putrefaction.—Abdomen : Stomach flaccid and of the middle size, contained the rice pudding undigested, with a small part of the skin of a prune; its mucous membrane of a light pink colour. The mesenteric glands were enlarged and numerous. The child had been fed occasionally with cow's milk.

Dr. Physick informed me that he had saved a child, affected in a similar way from eating a piece of pear. During the prevalence of the convulsions, the child not being able to swallow, a large quantity of tartar emetic and ipecacuanha was injected by a syringe into the stomach, through a catheter introduced down the throat: the piece of pear was thrown up by vomiting, and the child saved. He also told me that Mr. L. once lay in a stupor for several hours, from swallowing some article of food extremely difficult to digest: he also was relieved by its expulsion.

*Observation* 10*th.—Softening of the Stomach.*—In the summer of 1825, I dissected, for Dr. Otto, an infant child (about two years old) of Mrs. S. S., who was unwell for a week, without there being any well-marked symptoms of the nature of its disease. I found the left extremity of the stomach disorganised in all its coats, having been reduced to a light mahogany-coloured and pulpy state, about the consistence of a piece of glue soaked in cold water for twenty-four hours. Having no suspicion, at the moment, of such a state of the organ, I lacerated it inadvertently while only using a common force in drawing it upwards for examination. There was no undue redness in the mucous coat.

The brain of this child was large and somewhat congested, but not to a remarkable degree.

*Observation* 11*th.—Softening of the Stomach.*—Dr. La R. of this city, lost a child, in 1826, about four weeks old, in whom I found the stomach in a similar state of dissolution or softening. There had been, however, much pain in his infant, and flatulent tension of the abdomen.

These observations upon the stomachs of children, illustrate sufficiently the fact that the most extreme irritations of the stomach may exist during life, and may even be fatal, but yet not be manifested after death by unusual redness of the tissues affected. It would be indeed unphilosophical, and inconsistent with pathological observations on other parts, to exact from the stomach an invariable manifestation of disease, by redness and injection of its mucous surface after death. Let the redness of the skin in erysipelas be ever so strong during life, it frequently disappears wholly, by the retreat of the blood from its capillaries during death. Measles are similar in this respect, and the fact already mentioned is very worthy of attention, that if a fine injection be thrown into a subject who has died at the height of the eruption, the vessels originally dilated by the irritation manifest themselves by the greater quantity of injecting matter they receive: or, in other words, the eruption may be renewed after death, as I have satisfactorily ascertained by experiment. If any conjecture could be hazarded on these points, we are disposed to believe that, during the process of death, the vitality of parts in a state of inflammation is frequently so far diminished that they no longer have the power of attracting the fluids in undue quantity to them; consequently their redness disappears.

Acute inflammation of mucous tissues generally only thickens inconsiderably the part affected. It is attended by an increased secretion of mucus, of serum, of fibrino-mucous matter; and even an exhalation of red blood, from the blood-vessels being so extremely superficial. We see continually these phenomena going on in inflammation of the Schneiderian membrane and in colitis.

It has been seen that if an acute inflammation of the gastro-intestinal mucous membrane does not kill in its early stages, the injection with red blood is frequently by no means so great as it would have been in the case of an earlier death, and the changes which I have generally observed are as follow:—The stomach and bowels assume a dirty yellow or liquorice colour; the stomach presents internally small blotches of red at intervals, and frequently are found small filaments of coagulated blood adhering to the mucous coat of the stomach, seemingly at the orifices of the vessels from which they were discharged. The veins of the bowels are either partially or generally filled with blood. The intestinal mucus is abundant, adheres closely, and is tinged yellow by the bile. The mucous membrane of the stomach is easily peeled or scraped off by the finger-nail. The brain in this state most frequently exhibits marks of congestion, and with some inflammation of its meninges. The eyes and skin are yellow. In the skin there are apt to be left blotches of red blood, resembling purpura.

*Yellow Fever.*—In my notes of dissections performed on four yellow-fever patients, in the year 1820, I find the following remarks: In a female patient, aged forty, whose period of the disease I did not learn, the stomach contained a gill of black vomit, was of a natural thickness and texture, and presented over its whole mucous coat, in different regions, patches or blotches of extravasated blood. In a seaman belonging to the brig Martha, who had been sick at least nine or ten days, the stomach contained also black vomit, and was beset with small red points and spots of ecchymosed blood. In a third patient, a man aged forty, who complained slightly in the morning of one day, and was dead the next at seven o'clock A.M., I found the stomach extensively ecchymosed in its mucous coat, and its veins distended with blood; the black vomit was in great abundance in the stomach and intestines. In the fourth patient, a girl aged fourteen, who died on the fourth day of the disease, the mucous coat of the stomach and small intestine did not present the appearance of ecchymosis, but merely a minute arborescence of its veins.

*Observation 12th.—Acute Gastritis.*—During the month of July of the year 1826, I lost a patient, John Henderson, aged about sixty, from a violent remittent fever of about eight days' duration, attended with great prostration of strength, tenderness in the epigastrium, irritability of stomach, high fever, with coma and occasional delirium; a saffron-coloured skin and black tongue attended the last days of his disease. I examined him in the presence of Drs. Hodge and La Roche. The peritoneal surface of the abdomen, and of its viscera, we found healthy; but the mucous coat of the stomach presented, in its cardiac portion, a large motley patch of ecchymosed blood, as big as the palm of my hand; it also presented smaller patches of ecchymosis in other regions. His symptoms were, with the exception of black vomit, of such intensity, that his case would, in times of yellow fever, have been set down to that disease.

The same patched appearance sometimes is extended into the intestinal canal.

*Observation 13th.—Gastritis.*—May 30th, 1827, I dissected Edward Bloom, aged thirty-eight, a patient in the Alms-house. After a debauched course of life of some continuance, he was brought into the syphilitic ward for a sloughing ulcer of the penis, with mortification of the adjoining skin, without any well-marked sympathies, except a general prostration of his intellectual and physical powers: he died in five or six days after his admission.

Though there were no prominent gastric symptoms, as extreme irritability of the stomach, tenderness in the epigastrium, &c. we yet found the left end of the stomach, the jejunum, and the ascending colon, in a patched ecchymosed state, with here and there mahogany-coloured spots along the intestinal tube, arising from the collection of blood. The yellowish, semi-putrescent tinge, which the intestines under such circumstances are apt to have, was also present.

From the liability we are in to confound the redness of the stomach indicative of its inflammation during life, with the redness depending upon an obstruction to the circulation during the agonies of death, a principal object of the preceding pages has been to point out their difference, and to establish the important fact of gastric inflammation in most of those cases where the redness is observed. It is now proved that the redness arising from simple congestion, like the redness from injection, is uniformly diffused; whereas the redness from inflammation is generally partial and in patches, sometimes in the mucous membrane of the cardiac extremity of the stomach, and sometimes in that of the pyloric extremity.

Simple obstruction to the current of blood during the agonies of death could never produce such appearances; for, if it could, we ought to meet with them invariably. On the contrary, where an obstruction does occur, which I am disposed to believe is very uncommon in natural death, whether this obstruction arises from the liver or from the lungs, the arrest of blood should be manifested by an uniform tinge of red in the organs concerned, and not by patches here and there. Such marks of obstruction should also be more frequently seen, and better developed, in cases of enlarged indurated liver, in consumption, in dropsy of the thorax, and in all cases of obstruction of the lungs.

